# Neoadjuvant treatment does not influence PD-L1 expression in stage III non-small-cell lung cancer: a retrospective analysis of tumor samples from the trials SAKK 16/96, 16/00, 16/01, and 16/14*

**DOI:** 10.1016/j.esmoop.2023.101595

**Published:** 2023-07-11

**Authors:** D. König, S. Savic Prince, S. Hayoz, P. Zens, S. Berezowska, W. Jochum, E. Stauffer, V. Braunersreuther, B. Trachsel, S. Thierstein, M. Mark, S. Schmid, A. Curioni-Fontecedro, A. Addeo, I. Opitz, M. Guckenberger, M. Früh, D.C. Betticher, H.-B. Ris, R. Stupp, S.I. Rothschild, L. Bubendorf, M. Pless

**Affiliations:** 1 Department of Medical Oncology, University Hospital Basel, Basel; 2 Institute of Pathology and Medical Genetics, University Hospital Basel, Basel; 3 Swiss Group for Clinical Cancer Research, Bern; 4 Institute of Pathology, University of Bern, Bern; 5 Graduate School for Health Science, University of Bern, Bern; 6 Institute of Pathology, Centre Hospitalier Universitaire Vaudois (CHUV) and University of Lausanne, Lausanne; 7 Institute of Pathology, Cantonal Hospital of St. Gallen, St. Gallen; 8 Institute of Pathology, Promed, Marly; 9 Institute of Pathology, University Hospital Geneva (HUG), Geneva; 10 Department of Oncology, Cantonal Hospital of Graubünden, Chur; 11 Department of Medical Oncology, University Hospital of Bern (Inselspital), Bern; 12 Clinics of Medical Oncology, Cantonal Hospital of Fribourg (HFR), Fribourg; 13 Department of Oncology/Hematology, University Hospital Geneva (HUG), Geneva; 14 Department of Thoracic Surgery, University Hospital of Zurich, Zurich; 15 Department of Radiation Oncology, University Hospital of Zurich, Zurich; 16 Department of Medical Oncology/Hematology, Cantonal Hospital of St. Gallen, St. Gallen; 17 University of Bern, Bern; 18 Clinics for Thoracic Surgery, Hôpital du Valais, Sion, Switzerland; 19 Lurie Comprehensive Cancer Center, Northwestern University Feinberg School of Medicine, Chicago, USA; 20 Department of Medical Oncology, Centre Hospitalier Universitaire Vaudois (CHUV) and University of Lausanne, Lausanne; 21 Department of Medical Oncology/Hematology, Cantonal Hospital Baden, Baden; 22 Department of Medical Oncology, Cantonal Hospital Winterthur, Winterthur, Switzerland

**Keywords:** non-small-cell lung cancer, chemoradiation, immune checkpoint inhibitor, PD-L1 expression, predictive biomarker

## Abstract

**Background:**

The inclusion of immune checkpoint inhibitors (ICIs) in the treatment of operable stage III non-small-cell lung cancer is becoming a new standard. Programmed death-ligand 1 (PD-L1) protein expression on tumor cells has emerged as the most important biomarker for sensitivity to ICIs targeting the programmed cell death protein 1 (PD-1)–PD-L1 axis. Little is known about the impact of neoadjuvant treatment on PD-L1 expression.

**Patients and methods:**

We assessed PD-L1 expression by immunohistochemistry (Ventana SP263 assay) on tumor cells in treatment-naive diagnostic tumor samples and matched lung resections from patients with stage III non-small-cell lung cancer included in the Swiss Group for Clinical Cancer Research (SAKK) trials 16/96, 16/00, 16/01, and 16/14. All patients received neoadjuvant chemotherapy (CT) with cisplatin/docetaxel, either as single modality (CT), with sequential radiotherapy [chemoradiation therapy (CRT)] or with the PD-L1 inhibitor durvalumab (CT + ICI).

**Results:**

Overall, 132 paired tumor samples were analyzed from patients with neoadjuvant CT (*n* = 69), CRT (*n* = 33) and CT + ICI (*n* = 30). For CT and CRT, PD-L1 expression before and after neoadjuvant treatment did not differ significantly (Wilcoxon test, *P* = 0.94). Likewise, no statistically significant difference was observed between CT and CRT for PD-L1 expression after neoadjuvant treatment (*P* = 0.97). For CT + ICI, PD-L1 expression before and after neoadjuvant treatment also did not differ significantly (Wilcoxon test, *P* > 0.99). Event-free survival and overall survival for patients with downregulation or upregulation of PD-L1 expression after neoadjuvant treatment were similar.

**Conclusions:**

In our cohort of patients neoadjuvant treatment did not influence PD-L1 expression, irrespective of the specific neoadjuvant treatment protocol. Dynamic change of PD-L1 expression did not correlate with event-free survival or overall survival.

## Highlights

• Neoadjuvant CT, CRT, or immunochemotherapy does not influence PD-L1 expression in non-pathological complete remission patients.
• Potentially immune-sensitizing effects are unlikely to be caused by upregulation of PD-L1 expression.
• Change of PD-L1 expression after neoadjuvant treatment did not correlate with event-free survival or overall survival

## Introduction

Immune checkpoint inhibitors (ICIs) targeting the programmed cell death protein 1 (PD-1)–programmed death-ligand 1 (PD-L1) axis have become standard of care in both the treatment of the advanced/metastatic and the adjuvant/neoadjuvant setting for patients with non-small-cell lung cancer (NSCLC).^1^, ^2^, ^3^, ^4^, ^5^ In patients with operable stage III NSCLC, various clinical trials have investigated multimodal treatment strategies incorporating ICIs in the perioperative setting and many trials are still ongoing. We and others have reported promising results when PD-1/PD-L1 inhibitors were added to neoadjuvant chemotherapy (CT).^6^^,^^7^ The recently published phase III CheckMate 816 trial combined neoadjuvant chemotherapy with the PD-1 inhibitor nivolumab and demonstrated a significantly prolonged event-free survival (EFS) and time to death or distant metastases.^5^ Although a significant overall survival (OS) benefit has not yet been reported, combined immunochemotherapy will likely become a new treatment standard for patients with operable stage III NSCLC.

While PD-L1 expression detected by immunohistochemistry (IHC) is a required predictive biomarker in metastatic NSCLC for the selection of first-line PD-1 targeted monotherapy, the predictive role of PD-L1 in the neoadjuvant and adjuvant settings needs to be further defined. In the adjuvant treatment of patients with early-stage NSCLC, PD-L1 expression of ≥50% on tumor cells (TCs) predicts an OS benefit from treatment with the PD-L1 inhibitor atezolizumab.^8^ In the CheckMate 816 trial, PD-L1 expression was examined on baseline/pretreatment tumor tissue and a correlation with improved EFS was observed in patients across all PD-L1 expression subgroups, although the magnitude of benefit was higher in patients whose tumors had PD-L1 expression in ≥1% of TCs,^5^ indicating some degree of predictive potential of PD-L1 expression.

While different trials have demonstrated important changes in the tumor microenvironment as a result of neoadjuvant treatments, little is known about the impact of treatment modalities such as CT, radiotherapy (RT), and chemoradiation therapy (CRT) on PD-L1 expression, especially in the absence of an ICI. Preclinical studies provide the rationale for changes induced on PD-L1 expression, including activation of the Janus kinase/signal transducers and activators of transcription (JAK–STAT) pathway.^9^, ^10^, ^11^ However, published results from clinical trials have not yielded consistent results regarding the impact of different treatment modalities on PD-L1 expression. In patients with NSCLC, some studies reported that PD-L1 is upregulated by CT (upregulation),^12^^,^^13^ while others found that PD-L1 is downregulated by CT (downregulation).^14^^,^^15^ Likewise for CRT, downregulation of PD-L1 expression has been reported in one study,^16^ while another study reported upregulation of PD-L1 expression.^17^ Furthermore, results from preclinical studies indicate that RT might improve the sensitivity to subsequent PD-1/PD-L1 blockade.^18^, ^19^, ^20^ It is, however, not known whether this potential effect is mediated by changes in the PD-L1 expression.

In this study, we aimed to investigate whether PD-L1 expression is modified by the specific neoadjuvant treatment modality and whether alterations of PD-L1 expression predict response to neoadjuvant treatment and correlate with treatment outcomes. We therefore assessed PD-L1 expression by IHC on TCs in treatment-naive diagnostic tumor samples and matched lung resections from patients with operable stage III NSCLC who were treated in the Swiss Group for Clinical Cancer Research (SAKK) trials 16/96, 16/00, 16/01, and 16/14.^7^^,^^21^, ^22^, ^23^, ^24^ The SAKK trials investigated different neoadjuvant treatment strategies, including neoadjuvant CT, CRT, and CT followed by PD-L1 blockade.

## Patients and methods

### Patient cohort

Patients with operable stage III NSCLC enrolled in the four SAKK trials (i.e. 16/96, 16/00, 16/01, and 16/14) were included in this analysis. The detailed study designs, eligibility criteria, and methods of these trials have previously been published.^7^^,^^21^^,^^23^^,^^24^ In brief, all studies were designed for operable stage III NSCLC, both stage IIIA N2 and IIIB that could be encompassed in a single radiation port. The trials investigated different neoadjuvant treatments on a backbone of cisplatin/docetaxel CT (Supplementary Figure S1, available at https://doi.org/10.1016/j.esmoop.2023.101595). Patients in all trials received three cycles of neoadjuvant CT with cisplatin 100 mg/m^2^ and docetaxel 85 mg/m^2^, given once every 3 weeks. In the trials SAKK 16/00 (arm A) and 16/01, patients underwent a short course of preoperative accelerated RT (44 Gy in 22 fractions over 3 weeks) after completion of CT and prior to surgery. Patients in the trial SAKK 16/14 received two doses of the PD-L1 inhibitor durvalumab (750 mg/dose) 3 weeks after the last neoadjuvant CT (no RT). Surgery included an anatomical tumor resection with mediastinal lymph node dissection as previously described.^25^^,^^26^ The trials were conducted within the multicentric Swiss SAKK network. SAKK 16/00 additionally included participants from two European partner institutions. The studies were conducted sequentially: patients were enrolled from 1997 to 2000 (SAKK 16/96), from 2001 to 2012 (SAKK 16/00 and 16/01), and from 2016 to 2019 (SAKK 16/14). All studies were carried out in accordance with the principles of the Declaration of Helsinki and the guidelines on Good Clinical Practice. The protocols were approved by local ethics committees. Written informed consent was obtained from all patients.

### Study design

For the present retrospective translational analysis, we assessed PD-L1 tumor proportion score (TPS) expression by IHC on TCs in treatment-naive diagnostic tumor samples (preneoadjuvant treatment samples) and matched lung resections after neoadjuvant treatment (postneoadjuvant treatment samples) from patients enrolled in the aforementioned trials. Accordingly, we analyzed the impact of three different treatment modalities on PD-L1 expression: CT, CRT, and CT plus durvalumab (CT + ICI). Approval for this retrospective translational study was obtained by the local ethics committee (Zurich, Switzerland, BASEC-Nr. 2018-01441).

### Tumor samples and PD-L1 assessment

Hematoxylin and eosin slides and formalin-fixed, paraffin-embedded NSCLC tumor samples from preneoadjuvant treatment and matched postneoadjuvant treatment tumor samples were retrospectively collected from 18 Swiss centers involved in the aforementioned SAKK trials, where the probes had been stored between 8 and 23 years. From the 362 patients enrolled in the trials SAKK 16/96, 16/00, and 16/01, we obtained paired pre- and postneoadjuvant treatment samples from 102 patients, including 18 from SAKK 16/96, 73 from SAKK 16/00, and 11 from SAKK 16/01 (Supplementary Table S1, available at https://doi.org/10.1016/j.esmoop.2023.101595). For the more recent trial SAKK 16/14, PD-L1 results from matched pre- and postneoadjuvant treatment tumor samples were already available from 30 out of 67 patients (Supplementary Table S2, available at https://doi.org/10.1016/j.esmoop.2023.101595). From the other trials, hematoxylin and eosin slides were reviewed, and the appropriate corresponding formalin-fixed, paraffin-embedded tumor blocks were selected for PD-L1 IHC. At least 100 TCs were required for a specimen to be eligible for the study. Tumor sample processing and PD-L1 staining were carried out at the Institute of Pathology and Medical Genetics of the University Hospital Basel, Switzerland. The Ventana SP263 assay (Ventana, Tucson, AZ, USA) was used on the Ventana BenchMark immunostainer (Ventana) according to the manufacturer’s recommendations. PD-L1 testing in the trial SAKK 16/14 was carried out accordingly, with the exception of two pretreatment samples that were Papanicolaou-stained cytology specimens, fixed in Delaunay solution. PD-L1 testing on these cytology specimens was carried out by a laboratory-developed test using the SP142 antibody (Ventana; dilution 1/100) on the Leica Bond platform (Leica Biosystems, Wetzlar, Germany). The SP142 laboratory-developed test was validated against the SP263 assay with a high overall percent agreement between the two assays (data not shown).^7^ The PD-L1 testing was carried out and evaluated in 29 tumor samples at the Institute of Pathology, University of Bern, Switzerland. As the same assay (SP262) was used, we abstained from repeating PD-L1 staining in these samples for tissue preservation. Scoring of PD-L1 expression was based on the TPS and carried out by expert lung pathologists (SSP at the University Hospital of Basel and SB at the University of Bern). The TPS represents the estimated percentage (0%-100%) of TCs showing partial or complete membranous PD-L1 staining.^27^ PD-L1 expression levels were scored as continuous variable in <1%, 1%-4%, and 5% increments in tumors showing >5% staining. In addition, TPS values were subgrouped into the clinically relevant cut-offs (<1%, 1%-49%, and ≥50%; and <1%; and ≥1%).

### Outcomes

We correlated PD-L1 TPS (pre- and postneoadjuvant treatment expression levels and dynamic alterations after neoadjuvant treatment) with patient outcomes, including EFS, OS, nodal downstaging, and pathological complete remission (pCR) to neoadjuvant treatment. Definitions were consistent with the corresponding trials: EFS was the time from registration or randomization to objective tumor progression or relapse, secondary tumor (SAKK 16/00 and 16/14), or death due to any cause, whichever occurred first. OS was the interval from the date of registration or randomization to the date of death from any cause. Patients who did not experience an event were censored at the time of last known survival. pCR was ≥95% necrosis or fibrosis in the trial SAKK 16/96 and absence of TCs (0%) in all other SAKK trials.

### Statistical analyses

Patients’ characteristics were summarized by median and range for continuous variables and by frequency and proportion for categorical variables. For the comparison of PD-L1 values pre- to postneoadjuvant treatment Wilcoxon signed rank tests and for comparisons between groups, Wilcoxon rank sum tests were used. PD-L1 values were also categorized into PD-L1 <1% versus ≥1% and compared pre- to postneoadjuvant therapy using McNemar tests. Time-to-event endpoints were analyzed using the Kaplan–Meier method along with its corresponding 95% confidence interval (CI) based on the log–log approach. Between-group survival curves and rates were compared using the log-rank test and the Kaplan–Meier method at a specific time point, respectively.

Two-tailed tests with a significance level of 0.05 were used for all analyses. All statistical analyses were carried out using SAS version 9.4 (SAS Institute Inc., Cary, NC) and R version 4.1.0 (R Foundation for Statistical Computing, Vienna, Austria).

## Results

### Study population

**Figure 1:**
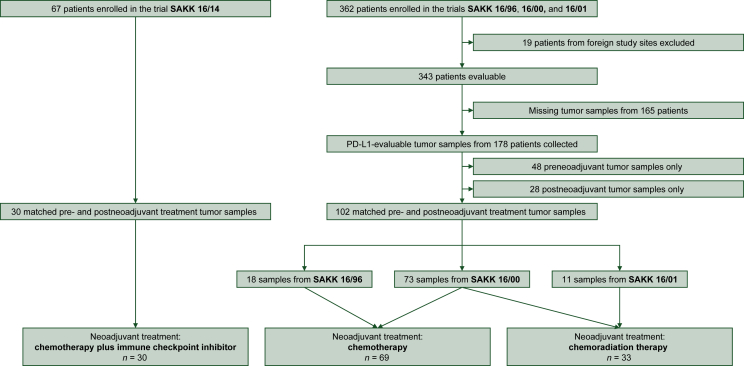
**Study overview.** PD-L1, programmed death-ligand 1; SAKK, Swiss Group for Clinical Cancer Research.

Demographics, disease characteristics, and outcome parameters of the patients with matched tumor samples from the trials SAKK 16/96, 16/00, 16/01, and 16/14 are summarized in Table 1, respectively. The parameters of the patient cohort were comparable to the corresponding overall trial population. The cohort of patients with matched tumor samples included 60 patients assigned to neoadjuvant CT (SAKK 16/96, 16/00 and 16/01) and 42 patients assigned to neoadjuvant CRT (SAKK 16/00 and 16/01). While all 102 patients had received at least one cycle of neoadjuvant CT, only 33 of the 42 patients assigned to CRT underwent RT within the study treatment protocol (median dose of 44 Gy). The cohorts of patients were therefore adapted: the CT cohort with 69 patients and the CRT cohort with 33 patients. All 30 patients in the CT + ICI cohort received the assigned treatment (Figure 1).

**Table 1:** Demographics, disease characteristics, and outcome parameters of patients in the trials SAKK 16/96, SAKK 16/00, SAKK 16/01, and SAKK 16/14

	CT + CRT cohort (*n* = 102)	Overall trial population (*n* = 362)
Trial, *n* (%)		
• SAKK 16/96	18 (17.6)	88 (24.3)
• SAKK 16/00	73 (71.6)	231 (63.8)
• SAKK 16/01	11 (10.8)	43 (11.9)
Neoadjuvant treatment strategy, *n* (%)		
• Neoadjuvant CT	69 (67.6)	202 (55.8)
• Neoadjuvant CRT	33 (32.4)	160 (44.2)
Eastern Cooperative Oncology Group performance status, *n* (%)		
• 0	70 (68.6)	239 (66.0)
• 1	31 (30.4)	122 (33.7)
• 2	1 (1.0)	1 (0.3)
Age (years)		
• Median (range)	59 (39-74)	60 (28-76)
Gender, *n* (%)		
• Female	32 (31.4)	110 (30.4)
• Male	70 (68.6)	252 (69.6)
Smoking status, *n* (%)		
• Current	43 (42.2)	126 (34.8)
• Former	54 (52.9)	208 (57.5)
• Never	5 (4.9)	27 (7.5)
• Missing data	0 (0)	1 (0.3)
Histological subtype, *n* (%)		
• Adenocarcinoma	36 (35.3)	132 (36.5)
• Squamous cell carcinoma	37 (36.3)	130 (35.9)
• Large cell carcinoma	9 (8.8)	33 (9.1)
• Poorly differentiated NSCLC	19 (18.6)	66 (18.2)
• NSCLC NOS	1 (1.0)	1 (0.3)
PD-L1 TPS expression on tumor cells^a^, *n* (%)		
• PD-L1 TPS <1%	47 (46.1)	NA
• PD-L1 TPS 1%-49%	36 (35.3)	NA
• PD-L1 TPS ≥50%	19 (18.6)	NA
Outcome parameters		
• Complete (R0) resection, *n* (%)	68/93^b^ (73.1)	230/286^b^ (80.4)
• Pathological complete remission, *n* (%)	0/93^b^ (0)	46/286^b^ (16.1)
• Event-free survival (months), median (95% CI)	12.7 (9.9-16.0)	12.3 (10.0-14.9)
• Overall survival (months), median (95% CI)	29.9 (21.7-40.3)	30.8 (24.4-40.3)

	CT + ICI cohort (*n* = 30)	Overall trial population (*n* = 67)
Eastern Cooperative Oncology Group performance status, *n* (%)		
• 0	20 (66.7)	52 (78)
• 1	10 (33.3)	15 (22)
Age (years)		
• Median (range)	60 (41-73)	61 (41-74)
Gender, *n* (%)		
• Female	15 (50.0)	32 (48)
• Male	15 (50.0)	35 (52)
Smoking status, *n* (%)		
• Current	13 (43.3)	28 (42)
• Former	16 (53.3)	36 (54)
• Never	1 (3.3)	3 (5)
Histological subtype, *n* (%)		
• Adenocarcinoma	19 (63.3)	37 (55)
• Squamous cell carcinoma	10 (33.3)	22 (33)
• NSCLC NOS	1 (3.3)	7 (10)
• Large cell carcinoma	0 (0)	1 (2)
PD-L1 TPS expression on tumor cells^c^^,^^d^, *n* (%)		
• PD-L1 TPS <1%	12 (40.0)	13 (19.4)
• PD-L1 TPS 1%-49%	13 (43.3)	26 (38.8)
• PD-L1 TPS ≥50%	5 (16.7)	20 (29.9)
• Missing	0 (0)	8 (11.9)
Outcome parameters		
• Complete (R0) resection, *n* (%)	28 (93.3)	51/55^e^ (92.7)
• Pathological complete remission, *n* (%)	0/30 (0)	10/55^e^ (18.2)
• Event-free survival (months), median (95% CI)	29.1 (13.6-NR)	NR (27.6-NR)
• Overall survival (months), median (95% CI)	NR (29.1-NR)	NR (NR-NR)

Patients with matched tumor samples (*left*) and the overall trial population (*right*).

CI, confidence interval; CRT, chemoradiation therapy; CT, chemotherapy; ICI, immune checkpoint inhibitor; NA, not applicable; NOS, not otherwise specified; NR, not reached; NSCLC, non-small-cell lung cancer; PD-L1, programmed death-ligand 1; SAKK, Swiss Group for Clinical Cancer Research; TPS, tumor proportion score.

a Assessment in the preneoadjuvant samples.

b Patients with tumor resection.

c Assessment in the preneoadjuvant samples for the matched samples.

d Assessment in either the preneoadjuvant or the post-neoadjuvant samples for the overall population (highest PD-L1 expression selected in patients with matched samples).

e Patients with tumor resection.

### PD-L1 expression

**Figure 2:**
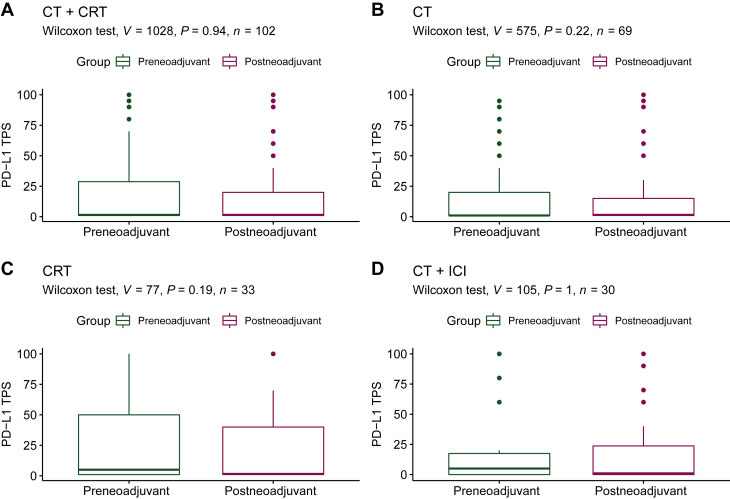
**Median programmed death-ligand 1 (PD-L1) expression [tumor proportion score (TPS)] in preneoadjuvant and postneoadjuvant treatment samples**. (A) Chemotherapy (CT) and chemoradiation therapy (CRT), (B) CT only, (C) CRT only, (D) CT + immune checkpoint inhibitor.

**Figure 3:**
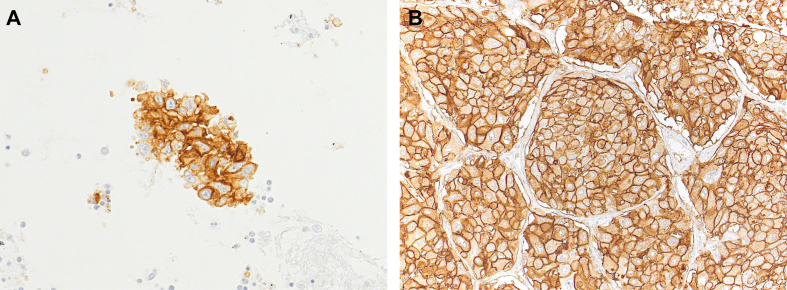
Example of a preneoadjuvant treatment sample [A; cell block, transbronchial fine-needle aspiration from lymph node, programmed death-ligand 1 (PD-L1) tumor proportion score (TPS) 100%] and a postneoadjuvant treatment sample (B; primary tumor/resection specimen, PD-L1 TPS 100%).

Of the 102 samples, the median PD-L1 TPS in the both pre-and postneoadjuvant treatment samples was 1% (<1%-100%; Supplementary Tables S3 and S4, available at https://doi.org/10.1016/j.esmoop.2023.101595). The preneoadjuvant treatment PD-L1 TPS was <1% in 47 samples (46%), 1%-49% in 36 samples (35%), and ≥50% in 19 samples (19%). There was significant intertrial heterogeneity regarding preneoadjuvant treatment PD-L1 expression from SAKK 16/00 versus 16/96 samples (*P* = 0.0083). This was not the case for the samples from SAKK 16/00 versus 16/01 (Supplementary Figure S2, available at https://doi.org/10.1016/j.esmoop.2023.101595). For all matched tumor samples, the PD-L1 TPS before and after neoadjuvant treatment did not differ significantly (Wilcoxon test; *P* = 0.94; Figure 2A). An example of a pre- and postneoadjuvant treatment sample is depicted in Figure 3. In the CRT cohort, the median PD-L1 TPS in the preneoadjuvant and postneoadjuvant treatment samples was 1%-4% (Supplementary Table S3, available at https://doi.org/10.1016/j.esmoop.2023.101595). In the CT cohort, the median PD-L1 TPS in samples prior to neoadjuvant treatment was <1% and thereafter 1%-4%. Statistically there was no significant difference in either group (CT and CRT) for the PD-L1 expression between the pre- and postneoadjuvant samples (Figure 2B and C). In the comparison of the treatment arms (CRT versus CT) there was no statistically significant difference for PD-L1 TPS after neoadjuvant treatment (*P* = 0.97; Supplementary Figure S3, available at https://doi.org/10.1016/j.esmoop.2023.101595), even with exclusion of the samples from SAKK 16/96 (Supplementary Figure S4, available at https://doi.org/10.1016/j.esmoop.2023.101595). These results did not change when PD-L1 expression levels were analyzed categorically with PD-L1 subgroups <1%, 1%-49%, and ≥50% (Supplementary Figure S5, available at https://doi.org/10.1016/j.esmoop.2023.101595). When categorized into PD-L1 TPS <1% versus ≥1%, we found a trend in the CRT cohort for PD-L1 TPS of <1% after neoadjuvant treatment (33.3% versus 48.5% pre- versus postneoadjuvant, respectively, McNemar test *P* = 0.059), whereas in the CT cohort, this trend was not observed (52.2% versus 43.5% pre- versus postneoadjuvant, respectively; McNemar test *P* = 0.359).

Of the 30 samples, the median PD-L1 TPS in the preneoadjuvant treatment samples was 5% (<1%-100%) and 1% (<1%-100%) in the postneoadjuvant treatment samples (Supplementary Table S3, available at https://doi.org/10.1016/j.esmoop.2023.101595). Preneoadjuvant treatment PD-L1 TPS was <1%, 1%-49%, and ≥50% in 12 (40%), 13 (43%), and 5 (17%) tumor samples, respectively. For the matched tumor samples, PD-L1 expression before and after neoadjuvant treatment did not differ significantly (Wilcoxon test; *P* > 0.99; Figure 2D). Categorized into PD-L1 TPS <1% versus ≥1%, no significant difference in postneoadjuvant treatment PD-L1 TPS <1% was observed (25.4% versus 17.9% pre- versus postneoadjuvant, respectively, McNemar test *P* = 0.20).

### Correlation with outcome parameters

**Figure 4:**
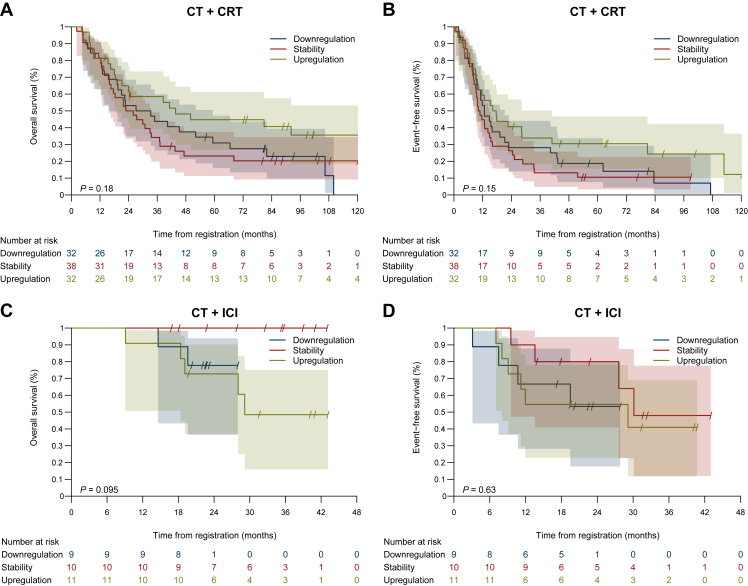
Overall survival and event-free survival of patients in the (A, B) chemotherapy and chemoradiation therapy (CT + CRT) cohort and (C, D) CT + immune checkpoint inhibitor (ICI) cohort (C, D) whose tumors had stability, upregulation, or downregulation of programmed death-ligand 1 expression after neoadjuvant treatment.

With a median follow-up time of 7.8 years (95% CI 7.1-8.6 years), the median EFS and median OS of the 102 patients in the CT/CRT cohort was 12.7 months (95% CI 9.9-16) and 29.9 months (95% CI 21.7-40.3), respectively (Supplementary Figure S6, available at https://doi.org/10.1016/j.esmoop.2023.101595). For both OS and EFS, upregulation of PD-L1 TPS after neoadjuvant treatment correlated with better patient outcomes, but the difference did not reach statistical significance (Figure 4A and B). Prolonged EFS and OS were observed in the few patients (*n* = 14) whose tumors had a PD-L1 upregulation from preneoadjuvant treatment TPS <1% to postneoadjuvant treatment TPS ≥1% compared with all other patients (median OS: 43.7 versus 27.1 months, *P* = 0.177 and median EFS: 22.3 versus 12.3 months, *P* = 0.275; Supplementary Figure S7, available at https://doi.org/10.1016/j.esmoop.2023.101595). No correlation was found for preneoadjuvant treatment PD-L1 TPS categorized into <1%, 1%-49%, and ≥50% with tumor downstaging after neoadjuvant therapy (TNM downstaging versus no downstaging; Supplementary Table S5, available at https://doi.org/10.1016/j.esmoop.2023.101595). Likewise, no correlation of preoperative PD-L1 TPS with pCR was found (Supplementary Figure S8, available at https://doi.org/10.1016/j.esmoop.2023.101595).

For the patients in the CT + ICI cohort, upregulation or downregulation of PD-L1 after neoadjuvant treatment had no significant impact on OS or EFS (Figure 4C and D).

## Discussion

We report the analysis of PD-L1 expression in matched tumor samples from patients with stage III NSCLC who underwent neoadjuvant CT, CRT, or CT + ICI in the four SAKK trials 16/96, 16/00, 16/01, and 16/14. Our analysis did not show a significant difference of the PD-L1 expression before or after neoadjuvant treatment, irrespective of the specific neoadjuvant treatment modality. Even in patients who had received a PD-L1 inhibitor during their induction regimen, there was no significant difference in PD-L1 expression in the surgical samples. It is thus unlikely in patients who did not achieve a pCR that there is a relevant effect of the described neoadjuvant treatment modalities on PD-L1 expression. Our results are in line with another study in a comparable patient and treatment setting in a single-center real-life cohort.^28^

Although the studied neoadjuvant treatments are nowadays either outdated (CT), have never been widely adopted in clinical practice (sequential CRT), or as for neoadjuvant immunochemotherapy—in view of the results from the phase III CheckMate 816 trial^5^ and the recently presented AEGEAN trial^29^—will likely be administered in a combined, rather than a sequential approach, the analysis of these specific neoadjuvant treatments allowed interesting conclusions:

First, we have shown that neoadjuvant CT did not influence PD-L1 expression. This finding is interesting in the context of the current adjuvant treatment landscape for stage III NSCLC. The results of the KEYNOTE-091 (PEARLS) study showed that patients who did not receive adjuvant CT had no benefit of the addition of pembrolizumab, which might suggest that there is some impact of CT on the biology of NSCLC or the host immune system that renders adjuvant ICI more efficacious.^30^ Our findings indicate that this effect is not mediated by CT-induced PD-L1 upregulation.

Second, our study suggests that the slightly increased pCR rate of combined neoadjuvant immunochemotherapy in patients with stage IIIA NSCLC with nodal involvement in the CheckMate 816 trial (pCR 22%) compared with the sequential approach in the SAKK 16/14 trial (pCR 18%) is not due to the influence of CT on the PD-L1 expression. If CT had induced a PD-L1 upregulation, the sequential combination of CT and durvalumab would have yielded a higher pCR rate in the SAKK 16/14 trial. The results from the SAKK 16/14 study are more comparable to the recently presented phase III AEGEAN trial that investigated perioperative treatment in patients with operable stage IIA-III NSCLC and included—in contrary to the SAKK 16/14 trial—a concurrent immunochemotherapy induction. pCR rate in both trials were comparable (18% versus 19% in stage IIIA patients in the AEGEAN trial).

Regarding the addition of RT to CT (sequential CRT) we did not observe an upregulation of PD-L1 expression compared with platinum-based CT alone. Our analysis seemed to indicate a downregulation of PD-L1 expression after CRT, similar to the results of a retrospective study in patients with stage II-III NSCLC who underwent neoadjuvant CRT.^16^ While the RT dosing in both trials was comparable (40-50 Gy versus 44 Gy in the SAKK trials), the SAKK trial investigated a sequential CRT approach, while patients from the study by Fujimoto et al.^16^ received concurrent CRT. Another trial in Japanese patients with stage III NSCLC who underwent concurrent CRT with a median RT dose of 60 Gy found an upregulation of PD-L1 expression in >90% of patients following neoadjuvant therapy.^17^ This study also included an RT-free cohort, whose tumor samples showed a trend for an upregulation of PD-L1 TPS after neoadjuvant treatment. It remains speculative whether the higher RT dose or the timing (sequential versus combined) influences the PD-L1 upregulation.

Our study cannot answer whether combined neoadjuvant procedures (CT with or without RT with or without ICI) might have an impact on PD-L1 expression. Interesting results are expected from the ongoing SAKK 16/18 trial (NCT04245514) that is investigating the addition of immune-modulatory RT combined with immunochemotherapy in patients with stage III NSCLC.

In our study, we were not able to demonstrate a prognostic information based on the alteration of PD-L1 TPS after neoadjuvant treatment. Neither upregulation nor downregulation of PD-L1 expression after neoadjuvant treatment resulted in any significant differences in EFS or OS compared with patients with stable PD-L1 expression, although we observed a trend toward better EFS and OS for patients with an upregulation of PD-L1 expression after neoadjuvant CT and CRT. However, this finding was not reproducible in the smaller cohort of patients with CT + ICI. Our observation of a trend for improved EFS and OS in patients with PD-L1 upregulation contrasts to the results published by Fujimoto et al.,^16^ who reported prolonged EFS and OS in patients with PD-L1 downregulation after CRT.^16^ Compared with our analysis, this study analyzed fewer patient samples and the EFS/OS results might have been influenced by the imbalance among the PD-L1 up-/downregulation cohorts. No prognostic information for change of PD-L1 TPS was found in the analysis of neoadjuvant CRT by Yoneda et al.^17^ The reason for these discordant observations remains unknown, likely influenced by the retrospective nature of all analyses, and possibly influenced by the specific PD-L1 assay. Importantly, the neoadjuvant regimen of the early SAKK trials did not include ICIs. We assume that only few, if any, patients included in the early SAKK trials received later-line ICIs. For the recent trial SAKK 16/14, the limited number of patients and the short follow-up make correlation of PD-L1 alterations with outcome parameters difficult.

Our study has several strengths and limitations. The strength arises from the standardized neoadjuvant treatment in a large homogenous trial population with the same CT backbone, allowing to draw conclusions about the impact of the specific treatment modality on PD-L1 expression. The analysis of samples from patients who had received additional RT (CRT) or durvalumab (CT + ICI) allowed interpatient and intertreatment comparison. To the best of our knowledge there have been no prospective trials addressing this issue, and most retrospective studies have assessed a single treatment modality, lacking a comparator arm. Our retrospective analysis included a high number of matched tumor samples and the largest set from patients with NSCLC. A limitation of our analysis is its retrospective character. Retrospective collection yielded paired samples of only 28% of all possible patients included in these series of trials. Reasons for the lack of paired samples was, first, failure to locate the samples of interest due to decentralized (local) storage of tumor samples in all but one trial (SAKK 16/14). In some patients the initial diagnosis had been established by cytology only, making it impossible to carry out adequate PD-L1 staining years later, or patients who progressed under treatment did not undergo subsequent surgical tumor resection, and lastly some patients achieved a complete or near-complete response to the neoadjuvant treatment with scarce residual tumor not allowing for further immunostaining procedures. Nevertheless, patient demographics, disease characteristics, and outcome parameters of our selected cohort were comparable to the corresponding overall trial population. Importantly, as there was no in-between biopsies during neoadjuvant treatment in the SAKK trials, we are not able to draw any conclusions regarding dynamic PD-L1 alteration (up-/downregulation) in patients who had pCR and thus—as we have shown previously—prolonged EFS and OS compared with non-pCR patients.^31^ To ensure homogeneity of PD-L1 IHC staining, all but a limited number of samples (*n* = 29, 22%) were centrally processed, stained, and analyzed, and an internationally validated PD-L1 staining (Ventana SP263 assay) was used for all cases, apart from two cytology samples that were analyzed with the Ventana SP142 assay. We observed significant PD-L1 heterogeneity between the samples from patients included in the trial SAKK 16/96 compared with those from the more recent trial SAKK 16/00, with a predominantly absent PD-L1 staining in samples originating from the SAKK 16/96. This raised the question of whether antigenicity is preserved in older samples. Given the lack of data concerning PD-L1 stability in archived tumor samples, we cannot exclude a loss of antigenicity in older, archived samples. However, an analysis of the KEYNOTE-010 trial suggested that archival samples are not associated with a loss of PD-L1 expression compared with newly collected samples.^32^ Furthermore, PD-L1 expression grouped according to established cut-offs (PD-L1 <1%, 1%-49%, and ≥50%) was comparable between the older trials (SAKK 16/96, 16/00, and 16/01) and the most recent trial SAKK 16/14. Our results did not change with exclusion of the samples from the oldest study (SAKK 16/96). An effect of intratumoral and intertumoral heterogeneity of PD-L1 expression on our results cannot be excluded. Discordance of PD-L1 expression between the preoperative samples (primary tumor) and the corresponding resection specimens has been described,^33^ as well as intratumoral heterogeneity for small tumor samples.^34^ Therefore our analysis included only representative probes and excluded very small samples with low TC numbers (<100). Whenever possible we analyzed PD-L1 expression in corresponding probes from the primary tumor. However, this was not always possible, and especially for the preneoadjuvant treatment samples many were from lymph nodes. Heterogeneity in PD-L1 expression between primary tumor and corresponding lymph nodes has been reported.^28^^,^^35^ In our analysis, we did not observe any significant differences when comparing corresponding samples from the primary tumors only with those from mixed samples (primary tumor/lymph node or vice versa). However, due to the low sample size these data must be interpreted with caution. The different origin of the samples ultimately reflects the situation in clinical practice.

### Conclusion

In summary, the studied neoadjuvant treatments (CT, sequential CRT, and sequential immunochemotherapy) had no effect on PD-L1 expression. Potential ICI-sensitizing effects of RT, CT, and CRT are unlikely to be caused by the upregulation of PD-L1 expression, at least in patients who did not achieve a pCR. Our results indicate that assessment of PD-L1 expression may be carried out before or after neoadjuvant treatment.
