# Supplementary material for: Neoadjuvant treatment does not influence PD-L1 expression in stage III non-small-cell lung cancer: a retrospective analysis of tumor samples from the trials SAKK 16/96, 16/00, 16/01, and 16/14
Source: ESMO Open. 2023 Jul 11;8(4):101595. doi: 10.1016/j.esmoop.2023.101595 (PMC10515281; doi:10.1016/j.esmoop.2023.101595)
Supplement: Supplemental Material [file mmc1.docx]

**Neoadjuvant treatment does not influence PD-L1 expression in stage III non-small cell lung cancer. Retrospective analysis of tumor samples from the trials SAKK 16/96, 16/00, 16/01 and 16/14.**

**Supplemental Material**

**Figures**

*Supplemental Figure 1.*

Study designs of the trials SAKK 16/96, 16/00, SAKK 16/01 and 16/14.

*Supplemental Figure 2.*

Median PD-L1 expression (Tumor Proportion Score, TPS) in pre-neoadjuvant treatment samples (pre-NAT) from the trials SAKK 16/96 vs. 16/00 (A) and the trials SAKK 16/01 vs. 16/00 (B).


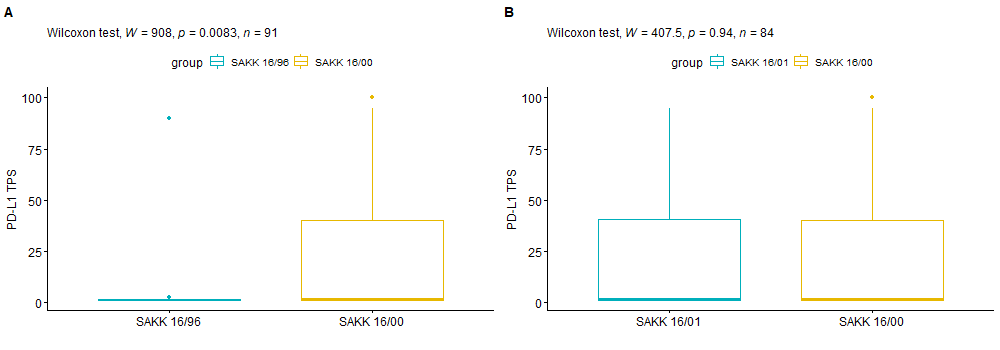


*Supplemental Figure 3.*

Chemoradiation (CRT) vs. Chemotherapy (CT). Median PD-L1 expression of the pre-neoadjuvant (pre-NAT) and the post-neoadjuvant treatment (post-NAT) samples.


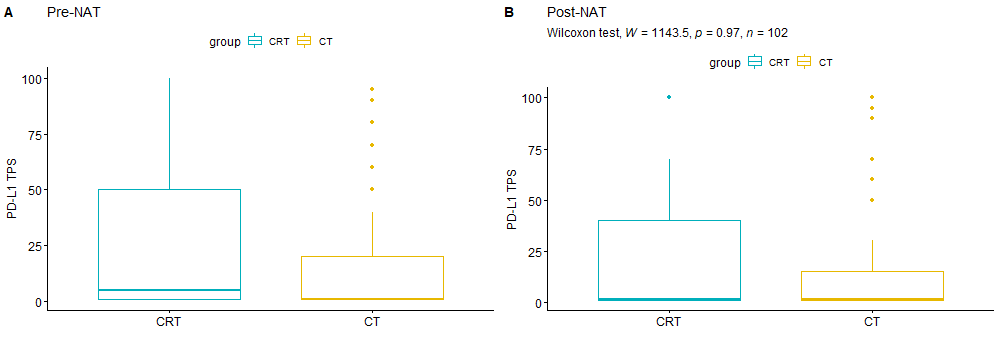


*Supplemental Figure 4.*

Chemoradiation (CRT) vs. Chemotherapy (CT) with exclusion of samples from the trial SAKK 16/96. Median PD-L1 expression of the pre-neoadjuvant (pre-NAT) and the post-neoadjuvant treatment (post-NAT) samples.


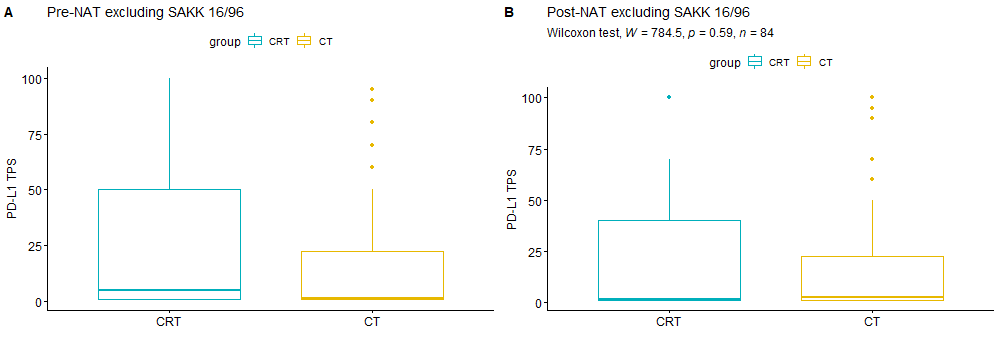


*Supplemental Figure 5.*

Pre- and post-neoadjuvant PD-L1 TPS for PD-L1 subgroups <1%, 1-49%, and ≥50% for Chemotherapy and Chemoradiation (CT+CRT) (A), Chemoradiation (CRT) (B), Chemotherapy (CT) (C) and Chemotherapy + Immune Checkpoint Inhibitor (CT+ICI) (D).


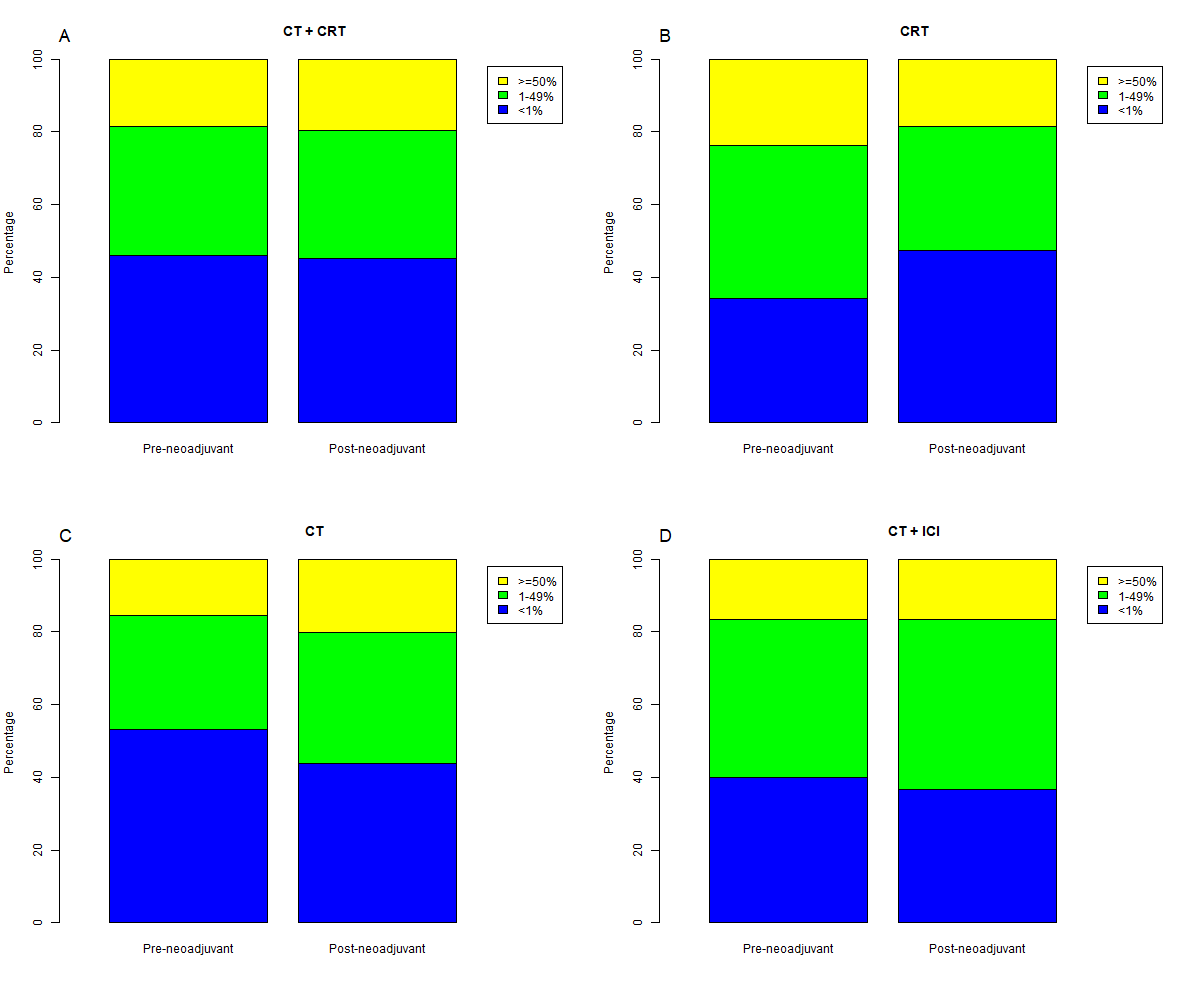


*Supplemental Figure 6.*

Overall Survival and Event-Free Survival in the Chemotherapy and Chemoradiation (CT+CRT) cohort (A, B) and the corresponding overall SAKK trial population (C, D).


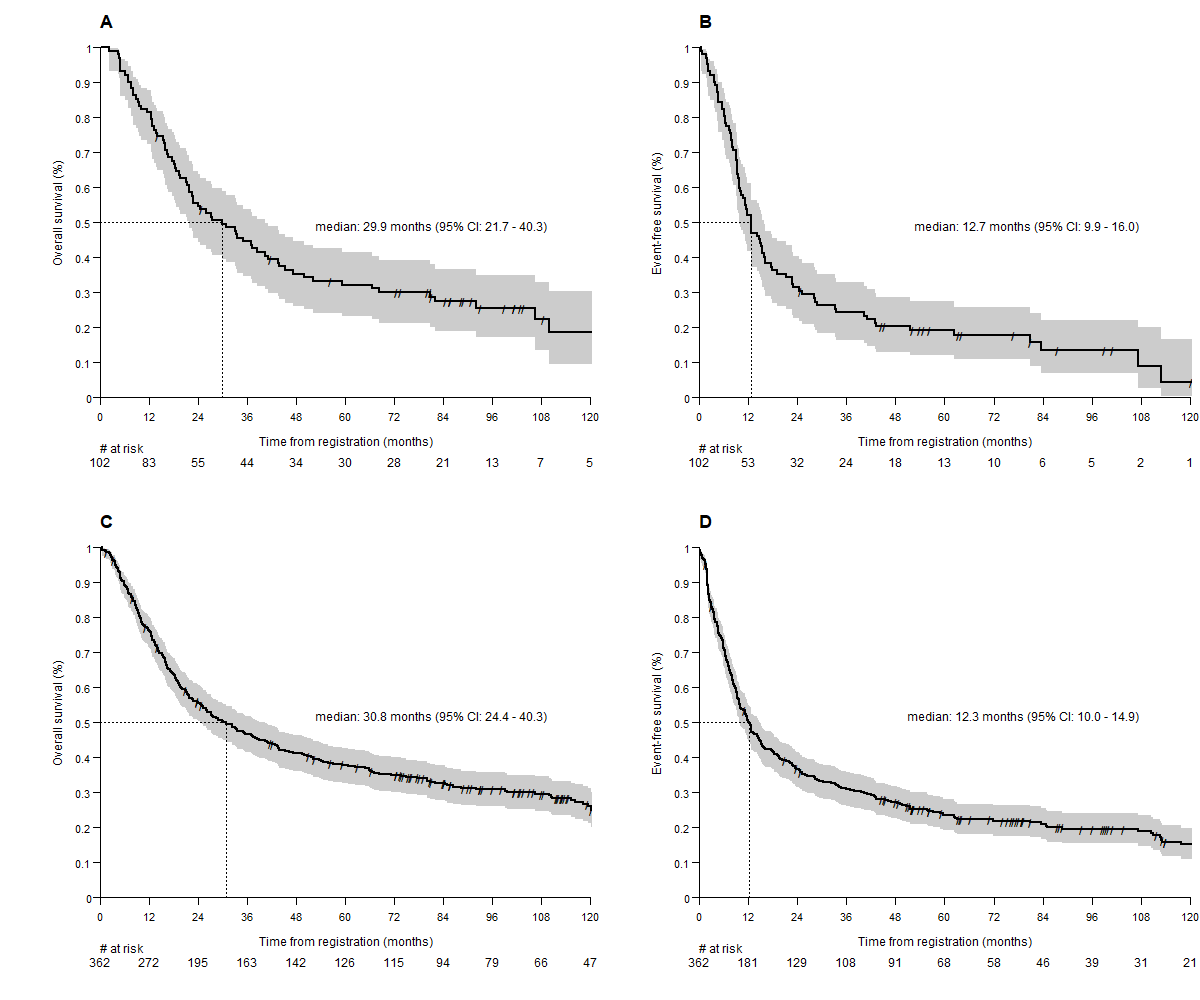


*Supplemental Figure 7.*

Overall Survival (A) and Event-Free Survival (B) in patients with pre-neoadjuvant treatment PD-L1 TPS <1% and upregulation after neoadjuvant treatment, vs. those patients without PD-L1 upregulation.


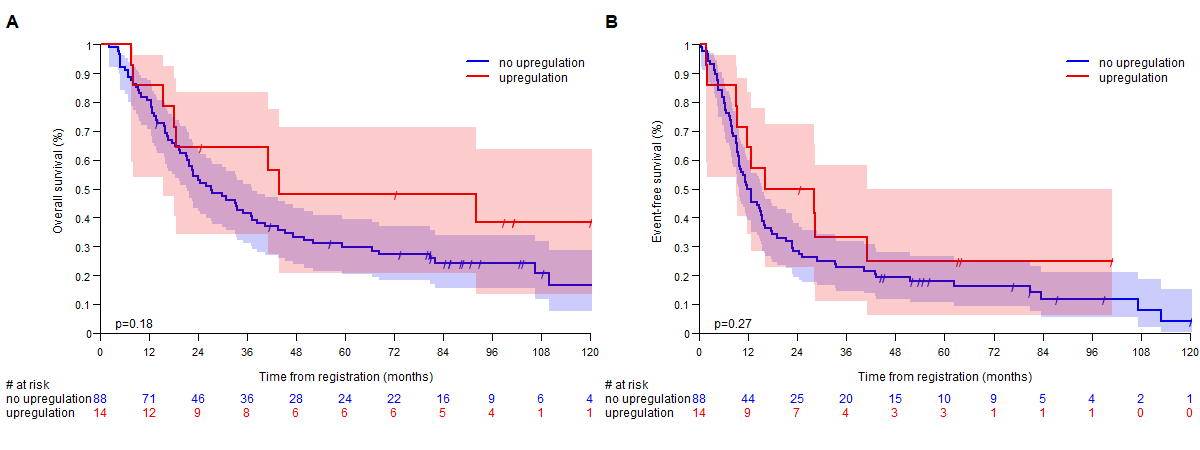


*Supplemental Figure 8.* Correlation of pre-neoadjuvant treatment PD-L1 TPS and pathological complete response (pCR).


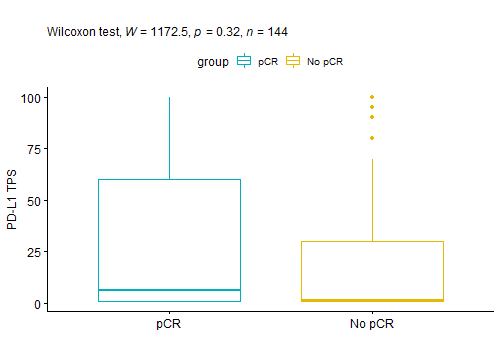


**Tables**

*Supplemental Table 1.*

Matched tumor samples from patients in the trials SAKK 16/96, SAKK 16/00 and SAKK 16/01 (Chemotherapy and Chemoradiation cohort).

| **SAKK trial** | **Patient Number** | **Neo-adjuvant Treatment Modality** | **Pre-NAT PD-L1 expression** (TPS) **on TC** | **Additional data** (Pre-NAT sample) | **Tissue origin**  (Pre-NAT sample) | **Post-NAT PD-L1 expression** (TPS) **on TC** | **Additional data**  (Post-NAT sample) | **Tissue origin**  (Post-NAT sample) |
| --- | --- | --- | --- | --- | --- | --- | --- | --- |
| 16/00 | 1 | CT | 70% |  | primary | 70% |  | primary |
| 16/00 | 2 | CT | 90% |  | primary | 70% |  | primary |
| 16/00 | 3 | CRT | 35% | discordant ^1^ | LN | 15% |  | LN |
| 16/00 | 4 | CT | <1% |  | LN | <1% |  | primary |
| 16/00 | 5 | CT | 40% | * | LN | 15% | * | primary |
| 16/00 | 6 | CT | 35% | discordant ^2^ | primary | 70% |  | primary |
| 16/00 | 7 | CRT | 95% |  | LN | 70% |  | primary |
| 16/00 | 8 | CRT | 40% |  | primary | <1% |  | LN |
| 16/00 | 9 | CRT | 20% |  | LN | 5% |  | LN |
| 16/00 | 10 | CT | <1% |  | primary | <1% |  | primary |
| 16/00 | 11 | CRT | <1% |  | LN | <1% |  | primary |
| 16/00 | 12 | CT | 95% | * | primary | 100% | * | primary |
| 16/00 | 13 | CRT | 10% |  | LN | 50% |  | primary |
| 16/00 | 14 | CRT | 60% |  | primary | 100% |  | primary |
| 16/00 | 15 | CRT | <1% |  | LN | <1% | multiple | primary, LN |
| 16/00 | 16 | CRT | <1% | * | LN | <1% | * | primary |
| 16/00 | 17 | CT | <1% |  | primary | <1% |  | primary |
| 16/00 | 18 | CRT | 40% |  | LN | 10% |  | primary |
| 16/00 | 19 | CRT | <1% |  | primary | <1% |  | primary |
| 16/00 | 20 | CT | <1% |  | primary | <1% |  | primary |
| 16/00 | 21 | CT | <1% |  | primary | <1% |  | primary |
| 16/00 | 22 | CRT | 1% |  | primary | <1% | multiple | primary |
| 16/00 | 23 | CT | 1% |  | primary | <1% |  | LN |
| 16/00 | 24 | CT | <1% |  | primary | 1-4% |  | primary |
| 16/00 | 25 | CT | <1% |  | primary | <1% |  | primary |
| 16/00 | 26 | CT | <1% |  | LN | 20% |  | primary |
| 16/00 | 27 | CRT | 70% | * | primary | <1% | * | primary |
| 16/00 | 28 | CT | 1% |  | primary | <1% |  | primary |
| 16/00 | 29 | CT | <1% |  | primary | <1% |  | primary |
| 16/00 | 30 | CT | 30% |  | primary | 15% |  | primary |
| 16/00 | 31 | CT | 20% | * | primary | 10% | * | primary |
| 16/00 | 32 | CT | 60% |  | primary | 95% |  | primary |
| 16/00 | 33 | CRT | 25% |  | LN | 1% |  | primary |
| 16/00 | 34 | CT | <1% |  | primary | 60% |  | primary |
| 16/00 | 35 | CRT | 1% |  | primary | <1% |  | primary |
| 16/00 | 36 | CT | <1% |  | primary | <1% |  | primary |
| 16/00 | 37 | CRT | 1% |  | primary | 1-4% |  | primary |
| 16/00 | 38 | CT | 90% |  | LN | 90% |  | primary |
| 16/00 | 39 | CT | 20% |  | LN | 5% |  | primary |
| 16/00 | 40 | CT | <1% |  | primary | 95% |  | primary |
| 16/00 | 41 | CRT | 1% |  | primary | 1% | discordant ^3^ | primary |
| 16/00 | 42 | CRT | 10% |  | primary | 1-4% |  | LN |
| 16/00 | 43 | CRT | <1% |  | primary | <1% |  | primary |
| 16/00 | 44 | CT | <1% |  | LN (c) | 1-4% |  | primary |
| 16/00 | 45 | CT | 50% |  | primary | 5% | discordant ^4^ | LN |
| 16/00 | 46 | CRT | 50% |  | primary | 40% |  | primary |
| 16/00 | 47 | CRT | 10% |  | primary | 5% |  | primary |
| 16/00 | 48 | CT | <1% |  | LN | <1% |  | LN |
| 16/00 | 49 | CT | <1% |  | primary | <1% |  | LN |
| 16/00 | 50 | CT | <1% | * | primary | 5% | * | primary |
| 16/00 | 51 | CT | <1% | * | LN (c) | <1% | * | primary |
| 16/00 | 52 | CRT | 15% |  | primary | <1% | multiple | primary |
| 16/00 | 53 | CT | 5% |  | primary | <1% |  | primary |
| 16/00 | 54 | CRT | <1% |  | primary | 1% |  | primary |
| 16/00 | 55 | CT | 1% |  | LN | 10% | discordant ^5^ | primary, LN |
| 16/00 | 56 | CRT | <1% | * | LN | <1% | * | primary |
| 16/00 | 57 | CRT | 40% |  | primary | 20% |  | primary |
| 16/00 | 58 | CRT | <1% |  | primary | <1% |  | primary |
| 16/00 | 59 | CRT | 1-4% |  | primary | <1% |  | primary |
| 16/00 | 60 | CT | 90% |  | LN | 100% |  | LN |
| 16/00 | 61 | CT | 2% | * | primary | 95% | * | primary |
| 16/00 | 62 | CT | 80% |  | primary | 50% |  | primary |
| 16/00 | 63 | CT | 90% |  | primary | 100% |  | primary |
| 16/00 | 64 | CT | <1% |  | LN | 1% |  | primary |
| 16/00 | 65 | CRT | <1% |  | primary | <1% | * | primary |
| 16/00 | 66 | CT | <1% | * | primary | <1% | * | primary |
| 16/00 | 67 | CT | <1% | * | LN | <1% | * | primary |
| 16/00 | 68 | CRT | 100% | multiple | LN (c) | 100% |  | primary |
| 16/00 | 69 | CT | 1-4% |  | primary | 15% |  | primary |
| 16/00 | 70 | CRT | 100% |  | primary | 70% |  | primary |
| 16/00 | 71 | CT | <1% |  | LN | 1-4% |  | primary |
| 16/00 | 72 | CT | 20% |  | LN | 25% |  | primary |
| 16/00 | 73 | CRT | 5% |  | LN | 35% | discordant ^6^ | primary |
| 16/01 | 74 | CRT | <1% | * | LN | <1% | * | primary |
| 16/01 | 75 | CRT | 1% |  | primary | 40% |  | primary |
| 16/01 | 76 | CRT | 90% | * | primary | 70% | * | primary |
| 16/01 | 77 | CRT | 95% |  | LN | 100% |  | LN |
| 16/01 | 78 | CRT | 80% | discordant ^7^ | LN | <1% |  | primary |
| 16/01 | 79 | CRT | <1% | * | primary | <1% | * | primary |
| 16/01 | 80 | CRT | 1% |  | primary | 1-4% |  | primary |
| 16/01 | 81 | CRT | <1% |  | LN | <1% | multiple | primary, LN |
| 16/01 | 82 | CRT | 1% |  | primary | 10% |  | primary |
| 16/01 | 83 | CRT | <1% |  | primary | <1% |  | LN |
| 16/01 | 84 | CRT | 1% |  | primary | 1% |  | primary |
| 16/96 | 85 | CT | 1% |  | LN | <1% |  | primary |
| 16/96 | 86 | CT | <1% |  | LN | <1% |  | primary |
| 16/96 | 87 | CT | <1% |  | LN | 30% |  | primary |
| 16/96 | 88 | CT | <1% |  | primary | <1% |  | primary |
| 16/96 | 89 | CT | 1-4% |  | primary | <1% |  | primary |
| 16/96 | 90 | CT | <1% |  | LN | <1% |  | primary |
| 16/96 | 91 | CT | <1% |  | LN | <1% |  | primary |
| 16/96 | 92 | CT | <1% |  | primary | <1% |  | primary |
| 16/96 | 93 | CT | <1% |  | primary | <1% |  | primary |
| 16/96 | 94 | CT | <1% |  | LN | <1% |  | primary |
| 16/96 | 95 | CT | 1-4% |  | LN | 1% |  | primary |
| 16/96 | 96 | CT | 1% |  | LN | <1% |  | primary |
| 16/96 | 97 | CT | <1% |  | LN | 1-4% |  | LN |
| 16/96 | 98 | CT | <1% |  | primary | <1% |  | primary |
| 16/96 | 99 | CT | <1% |  | LN | 1% |  | primary |
| 16/96 | 100 | CT | <1% |  | LN | 1-4% |  | primary |
| 16/96 | 101 | CT | 90% |  | LN | 100% |  | LN |
| 16/96 | 102 | CT | <1% |  | primary | 15% |  | LN |

PD-L1 staining performed with the Ventana SP263 assay (Ventana, Tucson, Arizona, USA), at the Institute of Pathology and Medical Genetics, University Hospital of Basel, Basel, Switzerland, and at the Institute of Pathology, University Hospital of Bern (Inselspital), Bern, Switzerland (29 samples marked with *).

Pre-NAT: Pre-Neoadjuvant Treatment; Post-NAT: Post-Neoadjuvant Treatment; TC: Tumor Cells; TPS: Tumor Proportion Score; CT: Chemotherapy; CRT: Chemoradiation; Primary: Primary Tumor; LN: Lymph Node; LN (c): Cell Block.

Discordant: tumor samples with discordant PD-L1 expression, all have been re-assessed and PD-L1 expression revised:

^1^ n=2 (PD-L1 30% and PD-L1 40%), revised PD-L1 expression: 35%

^2^ n=2 (PD-L1 60% and PD-L1 10%), revised PD-L1 expression: 35%

^3^ n=2 (PD-L1 1% and PD-L1 1-4%), revised PD-L1 expression: 1%

^4^ n=2 (PD-L1 <1% and PD-L1 10%), revised PD-L1 expression: 5%

^5^ n=2 (PD-L1 10% and PD-L1 1-4%), revised PD-L1 expression: 10%

^6^ n=2 (PD-L1 70% and PD-L1 <1%), revised PD-L1 expression: 35%

^7^ n=2 (PD-L1 80% and PD-L1 <1%), revised PD-L1 expression: 80%

Multiple: multiple tumor samples with the equal PD-L1 expression level.

*Supplemental Table 2*.

Matched tumor samples from patients in the trial SAKK 16/14 (Chemotherapy + Immune Checkpoint Inhibitor).

| **SAKK trial** | **Patient Number** | **Neo-adjuvant Treatment Modality** | **Pre-NAT PD-L1 expression** (TPS) **on TC** | **Additional data** (Pre-NAT sample) |  | **Post-NAT PD-L1 expression** (TPS) **on TC** | **Additional data**  (Post-NAT sample) |  |
| --- | --- | --- | --- | --- | --- | --- | --- | --- |
| 16/14 | 1 | CT-ICI | 5% |  |  | <1% |  |  |
| 16/14 | 2 | CT-ICI | 5% |  |  | 20% |  |  |
| 16/14 | 3 | CT-ICI | <1% | (c)* |  | <1% |  |  |
| 16/14 | 4 | CT-ICI | <1% |  |  | 5% |  |  |
| 16/14 | 5 | CT-ICI | <1% |  |  | <1% |  |  |
| 16/14 | 6 | CT-ICI | 20% |  |  | 70% |  |  |
| 16/14 | 7 | CT-ICI | <1% |  |  | <1% |  |  |
| 16/14 | 8 | CT-ICI | <1% |  |  | <1% |  |  |
| 16/14 | 9 | CT-ICI | <1% |  |  | 1% |  |  |
| 16/14 | 10 | CT-ICI | <1% |  |  | 1% |  |  |
| 16/14 | 11 | CT-ICI | <1% |  |  | <1% |  |  |
| 16/14 | 12 | CT-ICI | 5% |  |  | 40% |  |  |
| 16/14 | 13 | CT-ICI | <1% |  |  | 1% |  |  |
| 16/14 | 14 | CT-ICI | 80% |  |  | 60% |  |  |
| 16/14 | 15 | CT-ICI | 5% |  |  | 5% |  |  |
| 16/14 | 16 | CT-ICI | 5% |  |  | 60% |  |  |
| 16/14 | 17 | CT-ICI | 100% |  |  | 30% |  |  |
| 16/14 | 18 | CT-ICI | 1% |  |  | 1% |  |  |
| 16/14 | 19 | CT-ICI | 20% |  |  | 25% |  |  |
| 16/14 | 20 | CT-ICI | 20% |  |  | 5% |  |  |
| 16/14 | 21 | CT-ICI | 5% |  |  | <1% |  |  |
| 16/14 | 22 | CT-ICI | 10% |  |  | 1% |  |  |
| 16/14 | 23 | CT-ICI | <1% |  |  | 20% |  |  |
| 16/14 | 24 | CT-ICI | 100% |  |  | 1% |  |  |
| 16/14 | 25 | CT-ICI | 5% |  |  | <1% |  |  |
| 16/14 | 26 | CT-ICI | <1% |  |  | <1% |  |  |
| 16/14 | 27 | CT-ICI | 60% | (c)* |  | <1% |  |  |
| 16/14 | 28 | CT-ICI | 100% |  |  | 100% |  |  |
| 16/14 | 29 | CT-ICI | 5% |  |  | 90% |  |  |
| 16/14 | 30 | CT-ICI | <1% |  |  | <1% |  |  |

PD-L1 staining performed with the Ventana SP263 assay (Ventana, Tucson, Arizona, USA) at the Institute of Pathology and Medical Genetics, University Hospital of Basel, Basel, Switzerland. PD-L1 staining performed with the Ventana SP142 assay (Ventana, Tucson, Arizona, USA) for 2 samples (marked with *).

Pre-NAT: Pre-Neoadjuvant Treatment; Post-NAT: Post-Neoadjuvant Treatment; TC: Tumor Cells; TPS: Tumor Proportion Score; CT-ICI: Chemotherapy followed by immune-checkpoint inhibitor (Durvalumab); (c): cytology.

*Supplemental Table 3*.

PD-L1 protein expression (Tumor Proportion Score, TPS) on tumor cells in the Chemotherapy (CT), Chemoradiation (CRT) and Chemotherapy and Immune Checkpoint Inhibitor cohort (CT+ICI).

|  |  | |  | | |
| --- | --- | --- | --- | --- | --- |
| CT and CRT cohort (pooled) |  |  | |  |  |
| - Median PD-L1 expression (range) in the pre-neoadjuvant treatment samples |  | 1% (<1%; 100%) | |  |  |
| - Median PD-L1 expression (range) in the post-neoadjuvant treatment samples |  | 1% (<1%; 100%) | |  |  |
| CT cohort |  |  | |  |  |
| - Median PD-L1 expression (range) in the pre-neoadjuvant treatment samples |  | <1% (<1%; 100%) | |  |  |
| - Median PD-L1 expression (range) in the post-neoadjuvant treatment samples |  | 1-4% (<1%; 100%) | |  |  |
| CRT cohort |  |  | |  |  |
| - Median PD-L1 expression (range) in the pre-neoadjuvant treatment samples |  | 1-4% (<1%; 100%) | |  |  |
| - Median PD-L1 expression (range) in the post-neoadjuvant treatment samples |  | 1-4% (<1%; 100%) | |  |  |
|  |  | |  | | |
| CT+ICI cohort |  |  | |  |  |
| - Median PD-L1 expression (range) in the pre-neoadjuvant treatment samples |  | 5% (<1%; 100%) | |  |  |
| - Median PD-L1 expression (range) in the post-neoadjuvant treatment samples |  | 1% (<1%; 100%) | |  |  |
|  | | | | |  |

*Supplemental Table 4*.

Cross table of PD-L1 expression (Tumor Proportion Score, TPS) on tumor cells in matched tumor samples from the Chemotherapy and Chemoradiation cohort (n=102).

|  | **Post-neoadjuvant samples** (n = number of samples)  **PD-L1 expression level** | | | | | | | | | | | | | | | | |
| --- | --- | --- | --- | --- | --- | --- | --- | --- | --- | --- | --- | --- | --- | --- | --- | --- | --- |
| **Pre-neoadjuvant samples**  **PD-L1 expression level** | **PD-L1** | **<1%** | **1-4%** | **5%** | **10%** | **15%** | **20%** | **25%** | **30%** | **35%** | **40%** | **50%** | **60%** | **70%** | **90%** | **95%** | **100%** |
|  | **<1%** | 33 | 8 | 1 |  | 1 | 1 |  | 1 |  |  |  | 1 |  |  | 1 |  |
|  | **1-4%** | 8 | 5 |  | 2 | 1 |  |  |  |  | 1 |  |  |  |  | 1 |  |
|  | **5%** | 1 |  |  |  |  |  |  |  | 1 |  |  |  |  |  |  |  |
|  | **10%** |  | 1 | 1 |  |  |  |  |  |  |  | 1 |  |  |  |  |  |
|  | **15%** | 1 |  |  |  |  |  |  |  |  |  |  |  |  |  |  |  |
|  | **20%** |  |  | 2 | 1 |  |  | 1 |  |  |  |  |  |  |  |  |  |
|  | **25%** |  | 1 |  |  |  |  |  |  |  |  |  |  |  |  |  |  |
|  | **30%** |  |  |  |  | 1 |  |  |  |  |  |  |  |  |  |  |  |
|  | **35%** |  |  |  |  | 1 |  |  |  |  |  |  |  | 1 |  |  |  |
|  | **40%** | 1 |  |  | 1 | 1 | 1 |  |  |  |  |  |  |  |  |  |  |
|  | **50%** |  |  | 1 |  |  |  |  |  |  | 1 |  |  |  |  |  |  |
|  | **60%** |  |  |  |  |  |  |  |  |  |  |  |  |  |  | 1 | 1 |
|  | **70%** | 1 |  |  |  |  |  |  |  |  |  |  |  | 1 |  |  |  |
|  | **80%** | 1 |  |  |  |  |  |  |  |  |  | 1 |  |  |  |  |  |
|  | **90%** |  |  |  |  |  |  |  |  |  |  |  |  | 2 | 1 |  | 3 |
|  | **95%** |  |  |  |  |  |  |  |  |  |  |  |  | 1 |  |  | 2 |
|  | **100%** |  |  |  |  |  |  |  |  |  |  |  |  | 1 |  |  | 1 |

*Supplemental Table 5*.

Correlation of PD-L1 expression (Tumor Proportion Score, TPS) on tumor cells of the pre-neoadjuvant tumor samples with TNM downstaging from pre-neoadjuvant treatment staging to post-neoadjuvant treatment staging in the Chemotherapy and Chemoradiation cohort (pre-NAT TNM stage > post-NAT TNM stage) (n=94).

| **PD-L1 expression** | **No downstaging**  (n=41) | **Downstaging**  (n=53) |
| --- | --- | --- |
| PD-L1 <1% | 23 | 21 |
| PD-L1 1-49% | 11 | 22 |
| PD-L1 ≥50% | 7 | 10 |

(Pearson chi-square p=0.247).
